# Effects of Linagliptin and Pioglitazone on Fracture Healing in an Experimental Type 2 Diabetes Rat Model

**DOI:** 10.7759/cureus.32204

**Published:** 2022-12-05

**Authors:** Hamisi M Mraja, Sever Caglar, Muhammed Uslu, Bilal Yilmaz, Mustafa Fatih Dasci, Elif Yaprak Sarac, Metehan Demirkol

**Affiliations:** 1 Orthopaedics and Traumatology, Istanbul Florence Nightingale Hospital, Istanbul, TUR; 2 Orthopaedics and Traumatology, Health Sciences University, Bagcılar Training and Research Hospital, Istanbul, TUR; 3 Orthopaedics and Traumatology, Bİrecik State Hospital, Sanliurfa, TUR; 4 Orthopaedics and Traumatology, Health Sciences University, Bagcilar Training and Research Hospital, Istanbul, TUR; 5 Molecular Biology Genetics and Biotechnology, Istanbul Technical University, Istanbul, TUR; 6 Mechanical Engineering, Yıldız Technical University, Istanbul, TUR

**Keywords:** type 2 diabetes, streptozotocin, pioglitazone, linagliptin, bone healing

## Abstract

Aim: Our study aimed to examine the effects of Linagliptin, Pioglitazone, and their combination on fracture healing in a diabetes rat femur fracture model.

Material and methods: Type 2 diabetes mellitus (T2DM) induced rats were randomly divided into four groups: non-treated diabetes group (TD), Pioglitazone group (P), Linagliptin group (L), and Pioglitazone and Linagliptin group (PL). Daily oral dosage of pioglitazone (10 mg/kg/day), linagliptin (10 mg/kg/day), and their combination were administered. Femur fractures were stabilized intramedullary. At weeks 2 and 6, rats were sacrificed for evaluation radiologically, biomechanically, histopathologically, histomorphometrically, and immunohistochemically.

Results: Flexural strength of the L and PL groups were significantly higher compared to the P group. The highest healing score was in the L group and lowest in the P group, while the highest inflammation score was in the P group and lowest in the L group. A cluster of differentiation (CD) CD 34 reactivity was highest in the L group and lowest in the PL group.

Conclusion: Linagliptin treatment significantly increased histological healing scores, callus volume, biomechanical strength, and vascularity, however, minimized the inflammatory process, which was increased by pioglitazone. The combination of linagliptin and pioglitazone restored BMD and increased biomechanical strength. Linagliptin monotherapy is rarely indicated; hence, T2DM patients with a high risk of bone fractures can be considered for combined therapy of pioglitazone and linagliptin.

## Introduction

Diabetes mellitus (DM) is a metabolic disease with an increased incidence of fractures in both type 1 and type 2 DM (T2DM) patients, indicating diminished bone quality in the entire diabetes population [1,2]. DM also has an increased incidence of osteoporosis, possibly mediated by insulin deficiency and hyperglycemia in the bone [3,4]. Thus, DM impacts negatively on bone repair in response to the imbalance between bone formation and resorption, and the inflammatory dynamics [5].

Diabetes experimental animal models show slow fracture healing and fragile biomechanical properties with reduced cellular proliferation and collagen synthesis compared to non-diabetes animals [6]. Hyperglycemia impairs the response of osteoblast-like cells to insulin-like growth factor-I (IGF-1), potentially delaying bone formation [7]. Extended hyperglycemia produces high levels of advanced glycation end products (AGEs) hence bone healing process deteriorates [8].

Antidiabetic drugs affect bone metabolism [1-5]. Glycemic control is essential in the control of microvascular complications of T2DM. The main goal of treatment is principally achieved through drug combinations. Hence, lower doses of these combinations may be enough while managing normal glycemic control and minimizing any dose-related adverse events [9].

Dipeptidyl peptidase IV (DPP IV) is a serine protease expressed on the surface of most cell types. The mechanism of DPP IV inhibitors involves prolonging the half-life of glucagon-like peptide 1 (GLP-1) and gastric inhibitory polypeptide (GIP) and consequently promoting insulin secretion from β-pancreatic cells to improve glucose tolerance [10]. Many DPP IV substrates positively affect bone metabolism, suggesting a positive effect of DPP IV inhibition on the bone [11,12]. DPP IV inhibition has a reduced risk of bone fractures [13]. Linagliptin is one of the DPP IV inhibitors that stimulates insulin secretion, inhibits glucagon release after meals [14], and attenuates the loss of pancreatic β-cell mass and function associated with oxidative stress-induced inflammation [15].

Thiazolidinediones (TZDs) are peroxisome proliferator-activated receptor-r (PPAR-r) agonists and insulin sensitizers used to treat T2DM. They facilitate and strengthen the intracellular signaling cascade after insulin binds to its receptor [16]. Long-term use of TZDs is associated with bone loss and increased fracture risk in women with T2DM [17]. In addition, according to the recently reported study by Action to Control Cardiovascular Risk in Diabetes (ACCORD), the use of TZDs increases non-spine fracture in women with T2DM [18]. These results are related to decreased bone formation and increased bone resorption associated with TZDs [19].

In the clinic, DPP IV inhibitors and TZDs are one of the combination therapies in DM. Incretin-based treatments such as DPP IV inhibitors may protect against TZD-induced bone disorders. These two antidiabetic drugs have different mechanisms of action. This study evaluates the potential therapeutic effects of these drugs on the bone healing process. We aimed to examine the effects of a DPP IV inhibitor (Linagliptin), and a TZD (Pioglitazone), and their combination on fracture healing in a diabetes rat femur fracture model.

## Materials and methods

Animals

Seventy-two adult female age-matched Sprague-Dawley (240-290 g) rats were used in the study. Rats were obtained from the animal house of the University of Health Sciences Istanbul Bagcilar Training and Research Hospital. They were provided with free access to food and water under constant temperature (22˚C) and humidity (40%-60%) with a 12-h light and 12-h dark photoperiod. This study was approved by the Bagcılar Training and Research Hospital Ethics Committee (HADYEK 2019-22 No: 2019/98) for animal experiments. All procedures were performed following the regulations of ETS-123 and the European Convention for the Protection of Vertebrate Animals Used for Experimental and Other Scientific Purposes.

Experimental design

Rats were randomly divided into four groups (n=18): untreated diabetes group (TD), 10 mg/kg/day pioglitazone-administered diabetes group (P), 10 mg/kg/day linagliptin-administered diabetes group (L), and combination of pioglitazone and linagliptin-administered diabetes group (PL) [20,21]. Each drug was administered by gastric gavage daily for 14 or 42 days. A normal rat chow diet was assured to all rats during the study. Animals were checked daily for any signs of complications during the experimental period. No animals became severely ill or died before the experimental endpoint.

Establishment of diabetes model

Diabetes was induced by the intraperitoneal administration of 45 mg/kg streptozotocin (STZ) (Sigma®, Chemical Company, St. Louis, USA), dissolved in 0.01 M citrate buffer pH 4.5 [20,22]. Fasting blood glucose level was measured daily for three days. The accepted criterion for diabetes is a two consecutive days' measure of above 200 mg/dL of blood glucose. Diabetes rats received the drug treatment regimens orally throughout the study. Their body weights were measured and compared at study onset and weekly intervals. Animal recovery and activities were monitored daily every three to five days. Randomly, six rats of all groups were euthanized on the 14th day (early period) to determine the early effects of bone healing and the remaining 12 rats were euthanized on the 42nd day (late period). Radiographs were taken from all rats to assess the presence of bone repair or delayed union. The early period evaluation consisted of the radiologic, histopathological, histomorphometric, and immunohistochemical analyses while the late period evaluation additionally consisted of the biomechanical studies, including the 3-point bending test at the midshaft diaphysis.

Generation of fracture

All rats underwent an open fracture of the right femur with intramedullary stabilization using Kirschner (K)-wire. A transverse osteotomy was performed under ketamine (Ketalar®, Pfizer, Berlin, Germany) and xylazine (Rompun®, Bayer, Berlin, Germany) anesthesia. A transverse osteotomy was performed in the middle shaft of the femur to develop an open fracture using an electrical micro-saw. A1.2-mm K-wire was inserted anterogradely up to the intercondylar notch using an electrical driller motor. After open reduction, K-wire was retrogradely advanced up to the greater trochanter region. Radiographs were immediately taken to confirm fracture status and its reduction. Subcutaneous injection of 0.1 cc/kg carprofen (Rimadyl®, Zoetis, Parsippany, NJ, USA) was performed within 48 hr for pain control. On post-fracture day 14 and day 42, rats were euthanized by high-dose ketamine and xylazine anesthesia. All fractured right femurs were extracted, and after K-wires were cautiously removed, femurs were wrapped in gauze, immersed in formalin, and stored at -20˚C until analysis.

Micro-computed tomography

Specimens fixed in falcon tubes were scanned with SkyScan 1174v2 (Kontich- Belgium) computed tomography device. Each sample fixed in the chamber was scanned using a 0.25 mm Al filter at 50 kVp voltage, 800 μA current, and 40 W power. By optimizing the scanning values, 3,000 ms exposure time, 1,024x1,304 resolution, and 30.51 μm zoom factor were used. The right femurs of all subjects were scanned 360 degrees with 1.00-degree rotation for approximately 45 minutes each. After scanning, 360 raw images in TIFF format were reconstructed with the NRecon (Ver. 1.6.10.2) program, and 1024 cross-sections were obtained in BMP format (Bitmap image format).

Bone mineral density (BMD) calibrations were first applied to the dataset. These analyzes were performed using calcium densities of 0.25 g/mm^3^ and 0.75 g/mm^3^ CaHA calibration bar (phantom).

The images in BMP format were transferred to the CTAn (Ver. 1.16.4.1+) program. A circular area of ​​interest (region of Interest; ROI) was drawn semi-automatically in the horizontal plane on the sections to determine the area's boundaries to be measured. The histogram was evaluated to determine a threshold value by separating the sample's voxels and the surrounding air's voxels. The histogram determined the threshold value, with a black voxel indicating the minimum intensity as 0 and a white voxel indicating the maximum intensity as 255. Using the determined ROIs and threshold data, volumetric ratios were separately calculated. All data were transferred to the CTVol (Ver. 2.3.2.0) program, and three-dimensional modeling images of the samples were obtained.

Callus volume (CV), bone volume (BV), total volume (TV), and BV/TV ratio analyzes were calculated with CTAn (Ver. 1.16.4.1+) software using the ROI created on the fracture line on the images obtained after the scans.

Biomechanical testing

Three-point bending test was applied to determine the biomechanical properties of healed bone tissue. An electromechanical universal test device (Modified ALSA, Turkey) with Class 1 calibration with a load capacity of 3 kN was used. Femur specimens were placed horizontally on the measuring device with their anterior surfaces facing up. A vertical force was applied to reach the fracture line on a perpendicular axis. The bones were subjected to bending at 0.5 mm/s until a fracture occurred. The ultimate bending force (Fmax) was determined as the highest point of the force curve produced [23]. Fmax values were measured, and flexural strength (σbend) was calculated by using the following equations (Eqs 1-4): σbend=Mc/I (Eq. 1) M=FL/4 (Eq. 2) c=D/2 (Eq. 3) I=π(D4-d4)/64 (Eq. 4)

where M is the maximum bending moment, F is the force, L is the distance between supports, c is the distance to the neutral axis, I is the area moment of inertia, D is the outer diameter, and d is the inner diameter of the bone.

After the testing phase was completed, Fmax and σbend values were recorded for statistical analysis in Newtons (N) and megapascals (MPa), respectively.

Histopathological analysis

The histology of femoral fractures of all groups collected in the second and sixth week was evaluated by hematoxylin and eosin (H&E) and Masson's Trichrome staining. A scoring system was used to assess the callus healing. At least five randomly selected sections were scored for histological healing by a numerical scoring system of 1 to 10 points [24]. Inflammation was evaluated according to the lymphocyte infiltration by a scoring system; 0 for no inflammation, 1 for mild inflammation, 2 for moderate inflammation, and 3 for severe inflammation [25].

Histomorphometry analysis

For the histomorphometric analysis of the femoral fractures, the fracture areas were measured on 4-5 µm paraffin sections. Measurements included the cross-sectional area of bone and callus area consisting of fibrous tissue, cartilage, and ossifying tissue. At the end of the second week, the ratio of the cartilaginous callus area to total callus area (CAr, mm²) and the ratio of CAr, mm² to femoral bone diameter were calculated and given as %. Since the soft callus disappeared in the sections at the end of the sixth week, only the ratio of total callus diameter to femoral bone diameter was calculated and given as %.

Immunohistochemical analysis

Immunohistochemical evaluation was performed using the streptavidin biotin-peroxidase method. The monoclonal and polyclonal antibodies against Cluster of differentiation (CD) CD 34 and vascular endothelial growth factor (VEGF) proteins were used on the femoral sections. Five regions showing positive immunolabeling with relevant antigens were analyzed in terms of staining intensity by using semi-quantitative modified H-SCORE according to the literature [26]. Two researchers gave scores between 0 and 300 for five regions, and the scores were averaged. Femoral localizations of staining were determined, and the changes in protein expression and regional differences were detected.

Statistical analysis

All data are presented as mean ± standard deviation (SD). Statistical analyses of all findings were performed using GraphPad Instat 3.06 (GraphPad Inc, CA, USA). Normality was tested using the Kolmogorov-Smirnov test. Normally distributed variables were compared between two groups by using the Student t-test and between four groups by using one-way analysis of variance (ANOVA). Non-normally distributed variables were compared between two groups by Mann-Whitney U test, and between four groups by the Kruskal-Wallis H test. For post-hoc analysis, Tukey Kramer or Dunn’s multiple comparison tests were used. All the p-values of <0.05 were considered statistically significant.

## Results

Radiological findings

**Figure 1:**
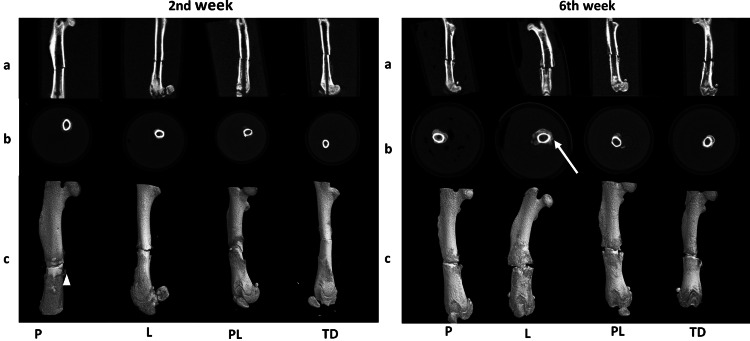
Micro-computed tomography images obtained in the second and sixth weeks. Non-treated diabetes group (TD), Pioglitazone group (P), Linagliptin group (L), and Pioglitazone and Linagliptin group (PL). Arrow head demonstrating soft callus and arrow demonstrating hard callus.

**Figure 2:**
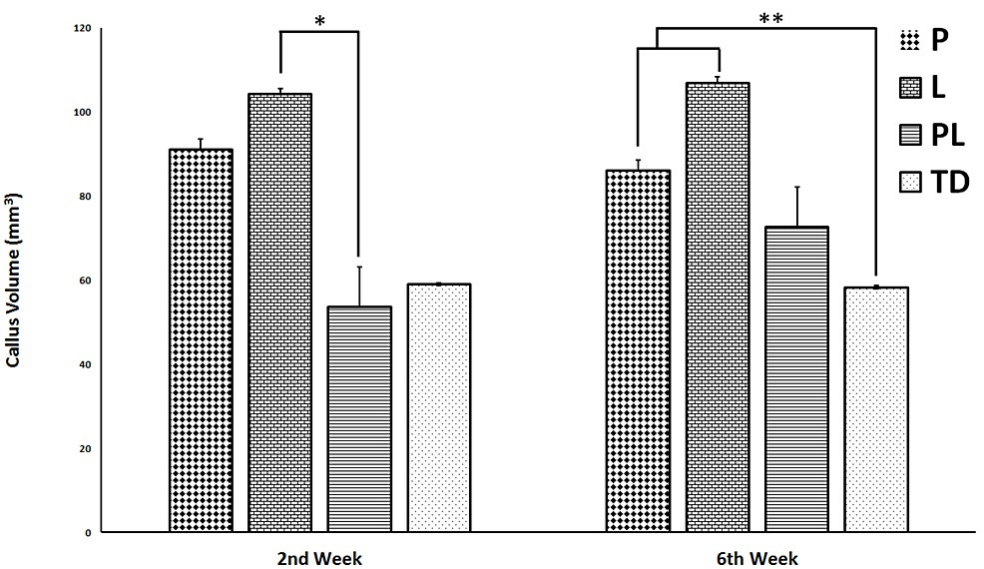
Radiologic evaluations of callus volumes calculated at 2 and 6 weeks. Y-axis presents the callus volumes of non-treated diabetes group (TD), Pioglitazone group (P), Linagliptin group (L), and Pioglitazone and Linagliptin group (PL). All data are represented as Mean ± SD. *L vs PL group p < 0.05; **P and L groups vs TD group p < 0.05.

At week 2, all the streptozotocin-induced diabetes rats had developed soft callus (Figure 1), and the CV increased significantly more in the L group by 94.3% compared to the PL group (p < 0.05). In addition, a noteworthy increase in the CV of the P group was detected radiologically (p ˃ 0.05). At week 6, the CVs of the P and L groups significantly increased compared to the CV of the TD group (p < 0.05) (Figure 2).

At week 2, the BV was found to be higher in P, L, and PL groups compared to TD groups, but there was no significant difference between all groups (p > 0.05). Also, at week 6, the BV was higher in L and PL groups than TD and P groups without any significance between all groups (p > 0.05).

At week 2, the BV/TV ratio of L group was higher than those of P, PL, and TD groups, still, no significant difference was found between all groups (p > 0.05). At week 6, although the BV/TV ratio was higher in P, L, and PL groups than those of TD group, there was no significant difference among the groups (p > 0.05).

**Figure 3:**
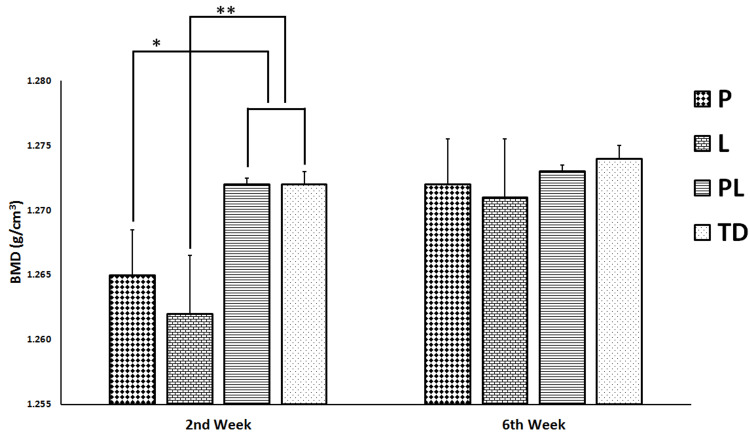
Radiological evaluations of bone mineral densities (BMD) at 2 and 6 weeks. Y-axis presents BMDs of non-treated diabetes group (TD), Pioglitazone group (P), Linagliptin group (L), and Pioglitazone and Linagliptin group (PL). All data are represented as Mean ± SD. *P group vs PL and TD groups p < 0.05; **L group vs PL and TD groups p < 0.05.

At week 2, the BMD value of the P group was significantly lower than those of PL and TD groups (p < 0.05) (Figure 3). Also, L group had a significantly lower BMD than those of PL and TD groups (p < 0.05). Nevertheless, BMDs of P and L groups showed no significant difference (p ˃ 0.05). At week 6, the intergroup analysis of BMDs showed no significant difference (p ˃ 0.05).

Biomechanical findings

**Figure 4:**
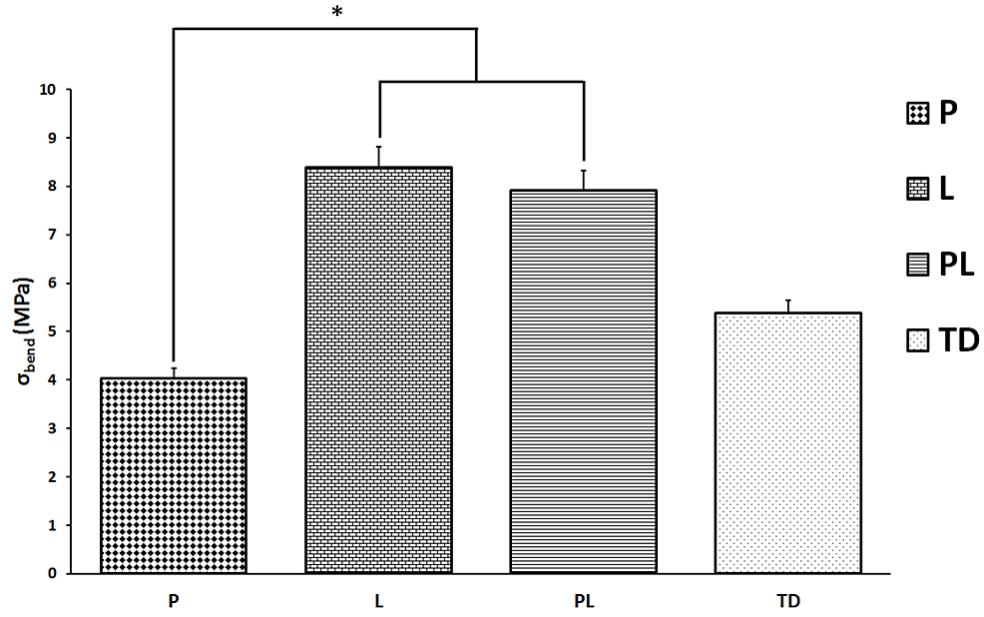
Biomechanical analysis of the flexural strength (σbend) value of healing bone at 6-week in non-treated diabetes group (TD), Pioglitazone group (P), Linagliptin group (L), and Pioglitazone and Linagliptin group (PL). *L and PL groups vs P group p < 0.05. MPa (Megapascal)

Biomechanical testing of the healing bone fracture was evaluated only at week 6. Flexural strength-σbend (MPa) values of L and PL groups were significantly higher than those of P group (p < 0.05) (Figure 4).

Histopathological findings

**Figure 5:**
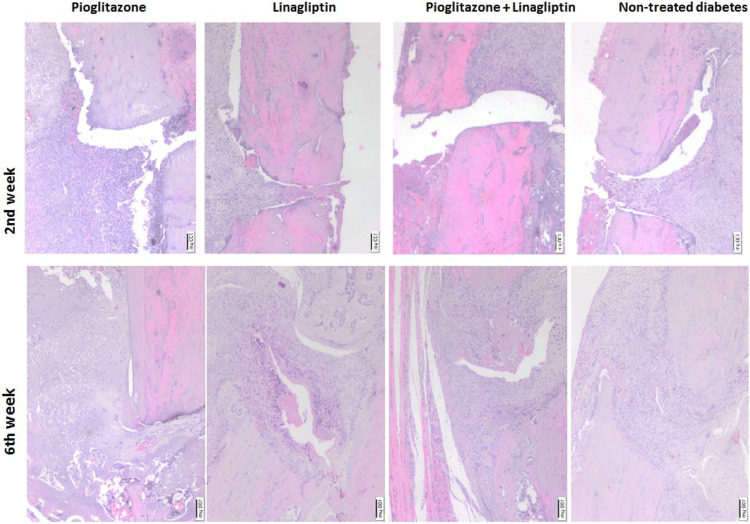
Histopathologic image showing the bone healing after 2 and 6 weeks of treatments in non-treated diabetes group (TD), Pioglitazone group (P), Linagliptin group (L), and Pioglitazone and Linagliptin group (PL). Hematoxylin and eosin staining (H&E, x40)

**Figure 6:**
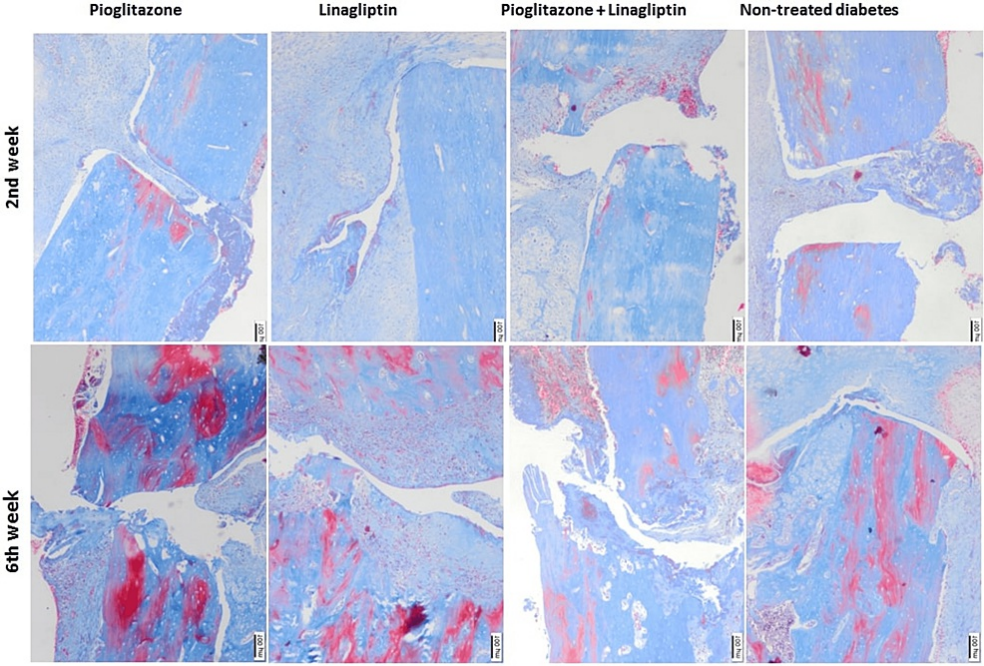
Histopathologic image showing the bone healing after 2 and 6 weeks of treatments in non-treated diabetes group (TD), Pioglitazone group (P), Linagliptin group (L), and Pioglitazone and Linagliptin group (PL). Masson trichrome staining (x40).

The H&E and Masson trichrome stainings are presented in Figures 5, 6. At week 2, large areas of soft callus consisting of cartilaginous callus could be differentiated with inflammation and infiltration. At week 6, a hard callus formed together with the cartilaginous callus whose diameter decreased in two dimensions. In addition, the increased immature bone tissues with a small amount of cartilage were found in some femoral regions, especially in L groups. However, the granulation tissues and local primary bone formations were noted in the femurs of some animals in P group. Moreover, it was observed that the fracture line did not disappear in most of the bone fractures at week 6. Inflammation was more pronounced in most rats at week 2 and persisted even at week 6.

Histological Healing Scores

**Figure 7:**
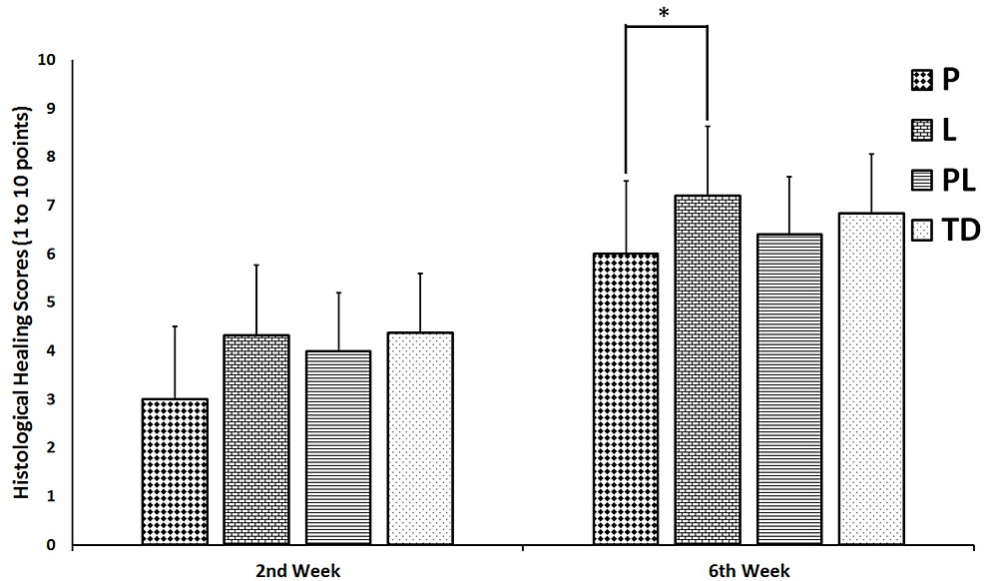
Histopathological evaluations of fracture healing scores calculated at 2 and 6 weeks of treatment in non-treated diabetes group (TD), Pioglitazone group (P), Linagliptin group (L), and Pioglitazone and Linagliptin group (PL). All data are represented as Mean ± SD. *P group vs L group p < 0.05.

The histological healing scores significantly increased for each group at week 6 compared to those at week 2 (p < 0.05) (Figure 7). However, there was no significant difference between the four groups regarding the histological healing scores at week 2 (p = 0.217). Moreover, at week 6, the highest histological healing score was found in L group and the lowest in P group. The histological healing score of P group was significantly lower than those of L group (p = 0.041) (Figure 7).

Inflammation Scores

**Figure 8:**
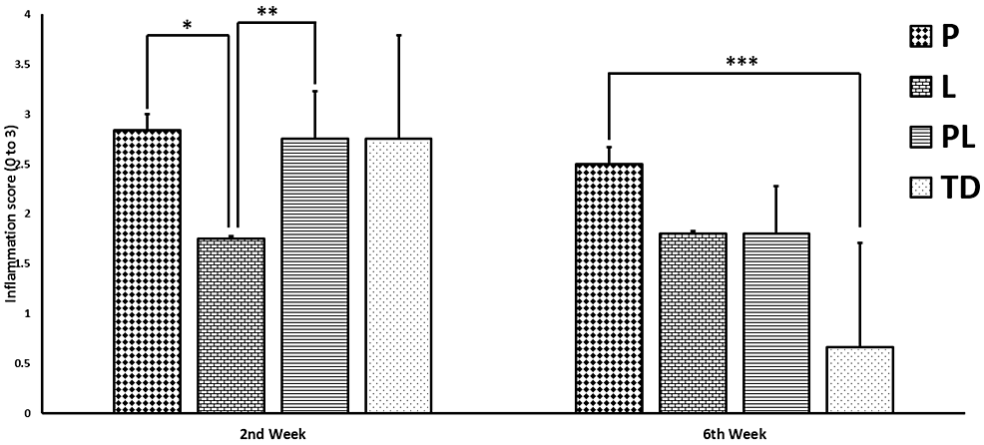
Histopathological evaluations of inflammations at 2 and 6 weeks of treatments in Non-treated diabetes group (TD), Pioglitazone group (P), Linagliptin group (L), and Pioglitazone and Linagliptin group (PL). All data are represented as Mean ± SD. *L group vs P group p < 0.05. **L group vs PL group p < 0.05. ***P group vs TD group p < 0.05.

The mean inflammation score of all groups at week 6 decreased compared to the second week (Figure 8). This decrease was significant, especially in the TD group (p = 0.0095). At week 2, the mean inflammation score of L group was lower than those of the other groups, while the mean scores of P and PL groups were significantly higher than those of L group (p = 0.0074). The highest inflammation score at week 6 was found in P group, and this value was significantly higher than those of TD group (p = 0.012).

Histomorphometric findings

At week 2, the cartilaginous callus area to total callus area ratio and the total callus diameter to femoral bone diameter ratio showed no significant difference between the four groups (p = 0.568 and p = 0.585, respectively). Similarly, at week 6, both ratios showed no significant difference between the four groups (p = 0.094 and p = 0.256, respectively).

Immunohistochemical findings

**Figure 9:**
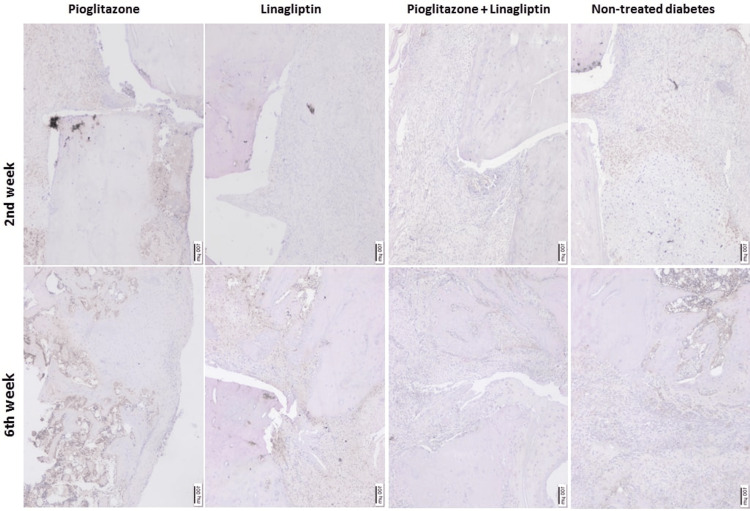
Microscopical images of cluster of differentiation (CD) CD 34 immunoreactivities detected at 2 and 6 weeks of treatments in non-treated diabetes group (TD), Pioglitazone group (P), Linagliptin group (L), and Pioglitazone and Linagliptin group (PL). (x40 magnification)

**Figure 10:**
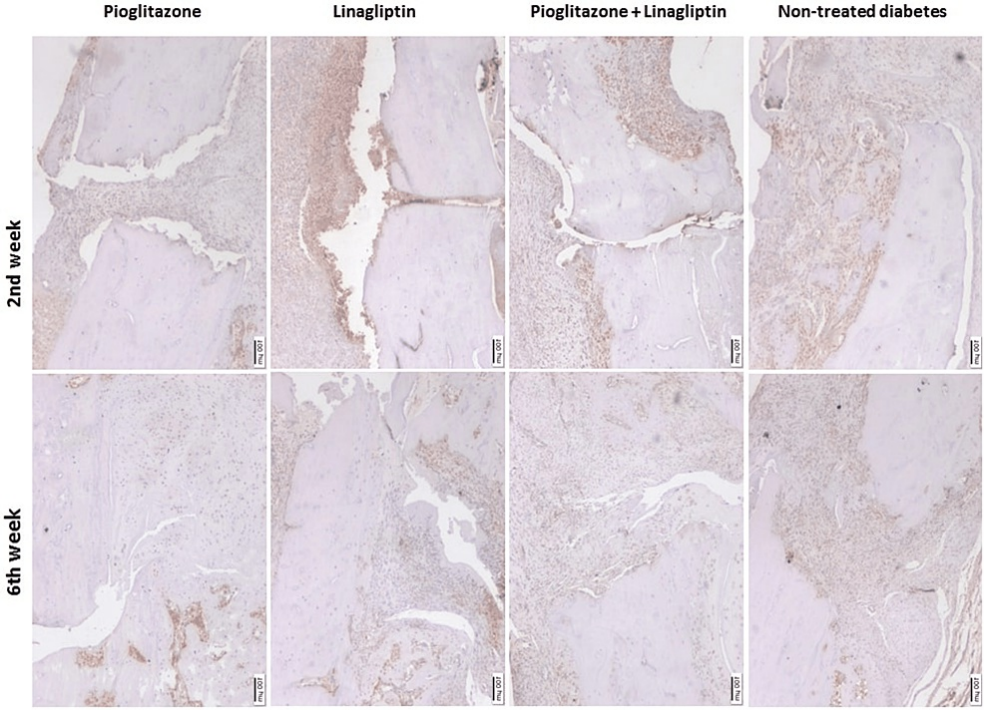
Microscopical images of vascular endothelial growth factor (VEGF) immunoreactivities detected at 2 and 6 weeks of treatments in non-treated diabetes group (TD), Pioglitazone group (P), Linagliptin group (L), and Pioglitazone and Linagliptin group (PL). (x40 magnification)

CD34 and VEGF immunoreactivity analyses are presented in Table 1 and the microscopical images are in Figures 9, 10. There was a decrease in the CD34 immunoreactivity at week 6 in TD, P, and PL groups compared to the immunoreactivities detected at week 2, without any significant difference (p > 0.05). The score of L group increased significantly in the late period compared to the early period (p = 0.048). At week 2, the highest CD34 immunoreactivity was found in P group, ands the lowest was found in PL group. The score of PL group was significantly lower than the score of P group (p = 0.029). Moreover, at week 6, CD34 reactivity was the highest in L group and the lowest in PL group. The score of PL group was significantly lower than the score of L group (p = 0.018) (Table 1).

**Table 1:** Immunohistochemical scores of CD34 and VEGF immunoreactivities in non-treated diabetes group (TD), Pioglitazone group (P), Linagliptin group (L), and Pioglitazone and Linagliptin group (PL). Data of H-SCORE were given as mean ± standard deviation. CD (Cluster of differentiation) VEGF (Vascular endothelial growth factor) *p < 0.05, PL group compared with Pioglitazone group. **p < 0.05, PL group compared with Linagliptin group.

CD34	Second week	Sixth week	P-value
P	120.0 ± 28.28	99.67 ± 54.80	0.446
L	62.6 ± 45.35	156.6 ± 71.71	0.048
PL	43.6 ± 19.03*	32.2 ± 43.73**	0.151
TD	73,5 ± 36,27	48,33 ± 29,94	0.286
P-value	0.029	0.018	
VEGF	Second week	Sixth week	P-value
P	160.0 ± 41.47	185.0 ± 42.78	0.331
L	226.0 ± 40.37	196.0 ± 47.22	0.316
PL	218.0 ± 26.83	157.0 ± 80.75	0.184
TD	223.5 ± 32.18	209.17 ± 22.00	0.454
P-value	0.921	0.591	

VEGF immunoreactivities in the late period decreased in L, PL, and TD groups compared to the scores in the early period. However, P group showed an increase in the late period compared to the early period without a statistical difference (p > 0.05). VEGF immunoreactivities showed no significant difference between the groups in early and late periods (p > 0.05) (Table 1).

## Discussion

In principle, untreated DM patients with bone fractures have increased risks of morbidity and mortality. Bone fractures are one of the morbidities. Diabetes causes loss of BMD which is one of the diabetes-related osteopenia challenges. Ultimately, bone fractures are uncontrollable. Appropriate medication for diabetes is essential in preventing these bone fractures.

Clinically, the combination therapy of DPP IV inhibitor and TZD is commonly used for diabetes management. Some studies have shown that these combination therapies have several advantages over monotherapies. Combination therapy of DPPIV inhibitor vildagliptin and a TZD pioglitazone in patients with T2DM was reported to reduce hypoglycemia risk and provide better glycemic control better compared to monotherapy [27]. Alogliptin/pioglitazone combination therapy was stated to improve β-cell function in patients with recently-onset T2DM [28]. Therefore, in the present study, we hypothesized that combination therapy including linagliptin and pioglitazone might help the process of bone fracture healing in diabetes patients. We found that the linagliptin treatment significantly increased histological healing scores, callus volume, biomechanical strength, and vascularity, however, minimized the inflammatory process, which was increased by pioglitazone. The combination of linagliptin and pioglitazone restored BMD and increased biomechanical strength.

A standardized osteotomized model of diaphyseal fracture reported in the literature was used in the present study [29]. The fracture was then fixed with a Kirschner wire [30]. A diabetes rat model commonly used to mimic diabetes-induced bone loss in human type 1-like bone loss was developed shortly after the injection of STZ [31]. This study is noteworthy for including both these two models and evaluating antidiabetic drugs in diabetes rats.

A previous study demonstrated that diabetes significantly also alters the trabecular microstructure, as indicated by the reduced BV/TV ratio of healed bones [6]. Linagliptin caused an increase in BV/TV ratio compared to the non-treated diabetes rats. The bone trabecula consists of an extracellular matrix and its collagen synthesis is impaired by diabetes. In the bone healing process, the callus has been reported to underdevelop its collagen levels by approximately 50% [6]. Hence, the trabecular development is diminished with the inadequate collagen content.

Large callus volumes and high BMD affect the bone healing process with a positive association [32]. Moreover, DM contributes to the deterioration of bone healing and BMD [1-5]. Micro-CT evaluation in the present study showed that both callus formation and fracture healing were better in the linagliptin-treated diabetes rats during the late period. However, both callus formation and fracture healing were poor with less BMD in the pioglitazone-treated diabetes rats during the late period. Irrespective of our theory that linagliptin could protect the deteriorating BMD in the healed bone of diabetes rats, linagliptin caused less BMD compared to both control rats and the rats having combination therapy. However, combination therapy resulted in a greater BMD than those of pioglitazone-treated diabetes rats, with a concomitant increase in restoring the flexural strength of healed bone.

The biomechanical findings of the present study showed that linagliptin with or without combination with pioglitazone developed a more rigid callus than those of only the pioglitazone group. Consequently, the augmented callus in linagliptin-treated diabetes rats was more resistant to the three-point bending test, with a 76.5% increase in the bony callus area and a 55.9% in flexural strength in comparison to those detected in non-treated diabetes animals. Therefore, linagliptin protects the poor bone healing process in diabetes by improving the biomechanical parameters at the late period of the healing process.

The fracture healing process requires revascularization of the bone [33]. Endochondral fracture healing involves angiogenic pathways, chondrocyte apoptosis, and cartilaginous degradation [34]. Bone repair site requires a well-organized vascularization system. Angiopoietin and VEGF pathways control this vascularization system [35]. In the healing process, VEGF is highly synthesized by hypertrophic chondrocytes and osteoblasts. Hence, these VEGF allow blood vessel formation and also the transformation of the avascular cartilaginous matrix into vascularized osseous tissue [33]. VEGF promotes both vasculogenesis and angiogenesis [36]. It also plays an essential role in the transition from cartilage to bone in endochondral bone formation [37] through enhanced osteoclastogenesis [38]. Hence, the VEGF is crucial in the neoangiogenesis and revascularization process at the fracture site, in addition to regulating bone remodeling. VEGF attracts endothelial cells, osteoblasts, osteoclasts [39], and autocrine regulation of chondrocyte function [37]. The immunohistochemical analysis of VEGF in the present study showed linagliptin treatment demonstrated no significant difference in vascularization or angiogenesis compared to the non-treated diabetes rats, suggesting a different effective pathway for fracture healing.

CD34, known as cell markers, is a transmembrane glycoprotein on endothelial cells, leukemic cells, and some progenitor cells. Its presence on endothelial cells of fracture callus and new bone was analyzed by immunohistochemistry of CD34. During the late period of the bone healing process, the linagliptin treatment resulted in a significant increase in CD34 reactivity in the fracture site. Despite the well-known poor vascularity in diabetes patients, Linagliptin provides a promising agent for stimulating bone healing by increasing vascularity in fractured bones.

To our knowledge, this is the first study investigating the effect of linagliptin and pioglitazone combination on bone healing in STZ-induced diabetes rodents according to the author's knowledge. Limitations of this study included not having a non-diabetes group to analyze linagliptin and pioglitazone effects on fracture healing as a control group.

## Conclusions

Our results indicate that linagliptin administration has an additive effect on fracture healing in diabetes rat models. This additive effect was enhanced by establishing mineralized tissue formation and improving the biomechanical properties. Also, it facilitated the development of a better callus and angiogenesis process of the newly formed bone. The combined therapy of pioglitazone and linagliptin allowed a biomechanical stronger healed bone with better BMD. Since linagliptin monotherapy is rarely indicated in DM treatment, pioglitazone and linagliptin combination protocol may be advantageous for bone healing in T2DM patients with a high risk of bone fractures.
